# Developing a national health research agenda for Lao PDR: prioritising the research needs of stakeholders

**DOI:** 10.1080/16549716.2020.1777000

**Published:** 2020-08-03

**Authors:** Dirk R. Essink, Kethmany Ratsavong, Esmee Bally, Jessica Fraser, Sengdavy Xaypadith, Manithong Vonglokham, Jacqueline Ew Broerse, Sengchanh Kounnavong

**Affiliations:** a Faculty of Science, Athena Institute, Amsterdam, Netherlands; b Lao Tropical Institute and Public Health, Lao Peoples Democratic Republic; c Department of Health Professional Education, Ministry of Health, Vientiane Capital, Vietnam

**Keywords:** LEARN: Sexual Reproductive Health, ANC and Nutrition, Lao People’s Democratic Republic, research needs, Delphi approach, health policy, mixed methods

## Abstract

**Background:**

Currently the health research system in Lao PDR is fragmented and largely donor led. Capacity among national public health institutes is limited to select priority research questions for funding.

**Objective:**

The objective of this capacity building and practice-oriented study is to describe the process and outcome of the first National Health Research Agenda for Lao PDR and how the agenda contributes to institutional capacity of the Ministry of Health, in order to contribute to evidence-informed public health policy making.

**Method:**

This activity used a mixed-methods approach. The overall design is based on principles of the interactive Learning and Action approach and consists out of 6 phases: (1) identification of needs, (2) shared analysis and integration, (3) nation-wide prioritization of research domains, (4) exploring specific research questions, (5) prioritization of research avenues, (6) dialogue and planning for action. The process involved interviews with experts in health policy and research (n = 42), telephone-based survey with district, provincial and national health staff (n = 135), a two-round Delphi consultation with experts in health policy and research (n = 33), and a workshop with policymakers, researchers, international organisations and civil society (n = 45) were held to gather data and conduct shared analysis.

**Results:**

11 research domains were identified and prioritised: Health-seeking behaviour; Health system research; Health service provision; Mother and child health (MCH); Sexual & reproductive health; Health education; Non-communicable diseases (NCDs); Irrational drug use; Communicable diseases (CDs); Road traffic accidents; Mental health. Within these domains over 200 unique research questions were identified.

**Conclusion:**

Our approach led to a comprehensive, inclusive, public health agenda for Lao PDR to realise better informed health policies. Questions on the agenda are action-oriented, originating in a desire to understand the problem so that immediate improvements can be made. The agenda is used within the MoH as a tool to fund and approve research.

## Background

Inequalities in health outcomes, access and use of health services, are a persistent problem in many countries. Evidence-informed public health policies and interventions can contribute to improving health outcomes and reducing inequities. Therefore, health research, addressing national and local health problems to support these policies, is critical in strengthening the health system. Prioritisation of research questions helps to ensure effective use of resources, makes research more needs based and increases the uptake of health research [1].

In Lao People’s Democratic Republic (Lao PDR) the research undertaken is often not specifically aligned with national priorities, and rarely commissioned by national health institutes [2–5]. In a review on the Lao PDR health system, Akkhavong et al. [2] outline the importance of health research and the need for capacity to translate this into practice. They highlight that health research is scarce and often disease oriented with limited focus on health system research. Further, they state that health research relies *on unpredictable donor support, which hampers evidence-based-policy- and decision-making* [2, p. 117]. This is further supported by Clarke et al. [6] who indicated that the reliance on donor support is one of the key constraints for evidence informed policy making in Lao PDR. These observations are explicitly recognised by the Ministry of Health in *the strategy on Promotion and Management of Health Research 2015–2020*. Defining research priorities is one of the six components of this strategy [5]. Here, they further state that research should contribute to the health-related objectives of the Millennium Development Goals, but no explicit reference to specific research topics and questions is made. Therefore, in this paper we present, and discuss the development of, the national public health research agenda in Lao PDR.

A research agenda serves to guide research and increases the likelihood that public health decision-making is based on evidence and subsequently, meet the needs of the population’s health and public health systems [7]. The research agenda itself provides a list of health topics which can then advance the translation of research into policies and action and provides a basis for better coordinating, leveraging, and identifying resources and activities to benefit the nation’s health [7,8]. A structured agenda is ‘flexible, systematic, transparent and replicable’ to improve priority-setting legitimacy [9]. This is particularly important in contexts where resources are scarce.

A recent review of prioritisation exercises led by the WHO illustrated ways to set research priorities, but stresses that approaches need to be context specific [10]. Examples range from expert consultations, literature reviews, Delphi techniques and economic evaluations, including program budgeting and marginal analysis, to citizen’s panels [1,10–15]. When the prioritisation of health research is done in a participatory manner, involving a range of stakeholders, it promotes alignment of academic and political interest with the needs of stakeholders, and research-derived evidence directed to the demands of local stakeholders is more likely to be used [11]. Therefore, a stakeholder engagement approach [1,16] was chosen to develop this agenda in a participatory manner.

The objective of this capacity building and practice-oriented study is to describe the process and outcome of the first National Health Research Agenda for Lao PDR and how the agenda contributes to institutional capacity of the Ministry of Health, in order to contribute to evidence-informed public health policy making.

## Study setting

This study was conducted in Lao PDR, a lower middle-income country situated in South East Asia with a population of under eight million. The health system comprises three administrative levels: 1) the central level; 2) the provincial level; and 3) the district level [2]. In 2013, the total research capacity comprised 1224 persons, an average of 1.8 persons per 10,000 population. The majority had either a master’s degree (542) or were senior medical doctors (608). Only 4% had a doctorate degree [5]. The two principal health research institutes are the Lao Tropical and Public Health Institute and the University of Health Sciences.

Despite improvements in reducing levels of mortality, child mortality, nutrition, non-communicable diseases (NCDs) and communicable diseases remain major health problems. NCDs account for 48% of the populations’ deaths and communicable, maternal, and nutritional issues account for 43% [17]. Also, health service provision, quality of services, and health finance support continue to fall short within the health system [17,18]. Thus, the improvement of health policies and health system reform needs to be a continued priority.

## Overall design, framework and approach

This process was conducted by the Lao Tropical and Public Health Institute (LaoTPHI), which is part of the Ministry of Health (MoH), in collaboration with the Vrije Universiteit Amsterdam.

To organise the agenda and give structure to the possible research questions, we have used Rudan’s organisation of a research agenda into domains, research avenues, and research questions [9,19]. Domains in this article refer to the larger research topics, and are mostly content oriented in the current study. Research avenues are subcategories of the domains and represent the specific research fields within the domains; they are part of the domains. The research questions are an operationalisation of specific avenues. By identifying the research questions, the aim of the research can be established, the methods needed for application can be planned, and the expected outcome can be measured and evaluated – thus making the agenda operational.

The overall emergent design is based on a checklist for priority setting [1] and the Interactive Learning and Action approach [16]. These approaches emphasise the need for a phased approach including preparation and inquiry, deciding and integrating priorities and a phase of translating priorities to practice. Both approaches are based on inclusiveness and co-creation.

The current mixed methods study consisted of six distinct, but related, research phases over two periods (April/July 2017 and February/July 2018). The six phases were: (1) identification of needs, (2) shared analysis and integration, (3) nation-wide prioritisation of research domains, (4) exploring specific research questions, (5) prioritisation of research avenues, (6) dialogue and planning for action.

The phases had their own specific methodological designs including selection criteria. The study included participants throughout Lao PDR, including health researchers, donors, governmental health policymakers and administrators at national, provincial and subnational level. All provincial and district health administrating bodies were included to ensure full geographical coverage of health administrators/practitioners.

**Figure 1.**
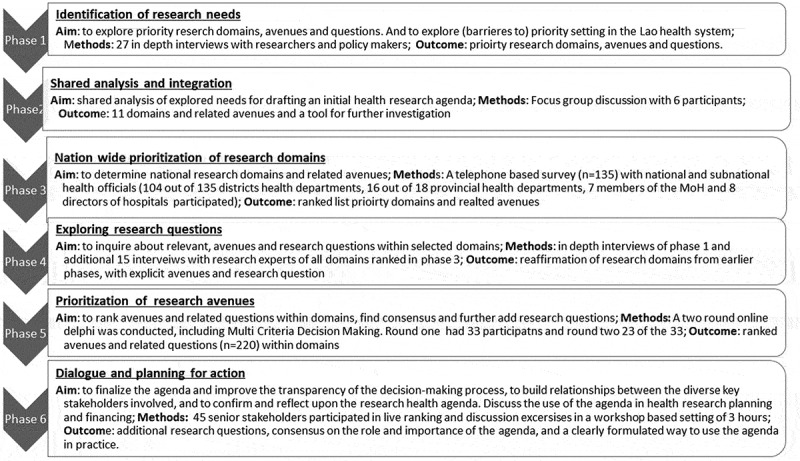
The six Phases of the agenda-setting process.

Describe the iterative process of developing the agenda, we have outlined the objectives, methods and results per phase separately in this paper. In Figure 1 we have summarised the phases which are subsequently explained in more detail in the paper.

## Phase 1: identification of needs

The aim of the first phase was to explore the research needs, based on research domains, avenues and questions, using key informant interviews with health researchers, policymakers, practitioners and program managers. A total of 27 face-to-face interviews of approximately one hour were held. Participant selection, based on purposive sampling, and recruitment was done by LaoTPHI/MoH. Policymakers and programme managers at national and sub-national level were included for their knowledge of national and local health priorities. Health researchers and practitioners (provincial and district hospitals) added valuable information by providing expert and up-to-date knowledge and practical experience (Table 1). The interviews were audio-recorded, transcribed and analysed in Max QDA. Interviews were conducted in English, when needed a translator was available to clarify questions and responses. Verbal informed consent was obtained from each participant before conducting and recording the interview. The outcome of these interviews was research themes and questions which were merged in a shared analysis in Phase two of the study. Data saturation was achieved on the level of domains.

**Table 1.** Characteristics of qualitative sample Phase 1.

Type of expert	Number
National level policymaker (MoH*)	3
Sub-national level policymaker (PHD/DHO**)	2
Vertical program manager	6
Technical staff member	3
Medical practitioner	4
University researcher (UHS**)	4
Researcher at Lao TPHI****	3
Researcher at PHD**	2
Total	27

*Ministry of Health; **provincial health department & District Health office; ***University of Health Sciences; ****Lao Tropical and Public Health Institute

## Phase 2: Shared analysis and integration

The aim of this Phase was to conduct shared analysis and prioritisation of topics generated in Phase one to further develop the agenda. Analysis was discussed in a workshop with six members including researchers from Vrije Universiteit Amsterdam, LaoTPHI and the Lao MoH. In the workshop, relevant domains and avenues were confirmed, merged (e.g. diabetes and cardiovascular diseases into NCDs) or re-labelled (e.g. health-seeking behaviour).

We downsized from 22 topics to 11 domains for the ranking exercise that covered each of the topics. The selected domains were as follows: road traffic accidents, communicable diseases, mental health, maternal and child health, irrational drug use, health system research, health education, service provision, sexual health, non-communicable diseases, health-seeking behaviour.

## Phase 3: nation-wide prioritisation of research domains

The objective of this phase of the study was to determine national health research priorities by asking (sub-)national health officials to rank the selected domains. To obtain full geographical coverage, a telephone-based survey in Lao language was conducted among *all* District Health Offices (DHOs; n = 146), Provincial Health Department (PHDs; n = 18), provincial hospitals (n = 12) and the heads of departments of the Ministry of Health (MoH; n = 6). These respondents were targeted as they are capable to reflect on public health research needs in their administrative areas and from their professional experience in the health sector.

A quantitative tool for ranking domains and associated avenues was developed using the results from Phases one and two (supplementary files). The tool consisted out of socio-demographic information and questions regarding priority-setting. Respondents were presented a prioritisation exercise in the form of a matrix. The paired-based-ranking-matrix was based on the multi-attribute utility theory and tools [20]. The eleven domains were presented on top of the matrix and the same eleven topics were set out on the left side of the matrix. The participant was asked to weigh each domain against one of the other domains included in the exercise. Consequently, the participant continuously weighs two domains and selects the one which has priority over the other. For example we asked; ‘which topic needs more priority for research in Lao, research to reduce irrational drug use or research to respond to NCDs’. The question was asked for each pair of domains and in total, the participant makes 55 choices which were scored by the researcher administering the interview. The results of these led to the prioritisation of the domains. We checked consistency of answering among respondents [21].

Data were recorded in Excel and analysed in Stata. In total, 140 questionnaires were filled in (response rate 76.9%). Overall the consistency of answers was high, only five respondents had more than one inconsistency within 55 paired rankings. After adjusting for inconsistencies, a total sample of 135 questionnaires was included in the study; 104 (77.0%) were director or deputy director of a DHO, 16 (11.9%) were director or deputy director of a PHD, eight (5.9%) were director or deputy director in a provincial hospital, and 7 (5.2%) represented a department of the MoH. The median age was 50 (IQR 47–54) and 70% were male. The majority of participants worked in urban areas (80.0%) compared to areas classified as rural (20.0%). Most participants highest obtained degree was a bachelor’s (44.4%), followed by participants with a master’s degree or higher (33.3%). About a quarter of participants had a background in research, mainly as a principle investigator (65.8%).

When a respondent consistently preferred one of the eleven domains above all other domains in the exercise, dominance of this specific domain occurred. Pooling all exercises together, the maximum number of times a domain can be prioritised above all other domains is 1350. The maximum count per domain is therefore 1350. To have a better understanding of how domains relate in the ranking, a ratio was used. The ratio indicates the proportion of the maximal count, with a maximum of 1.0 which equals 1350.

**Figure 2.**
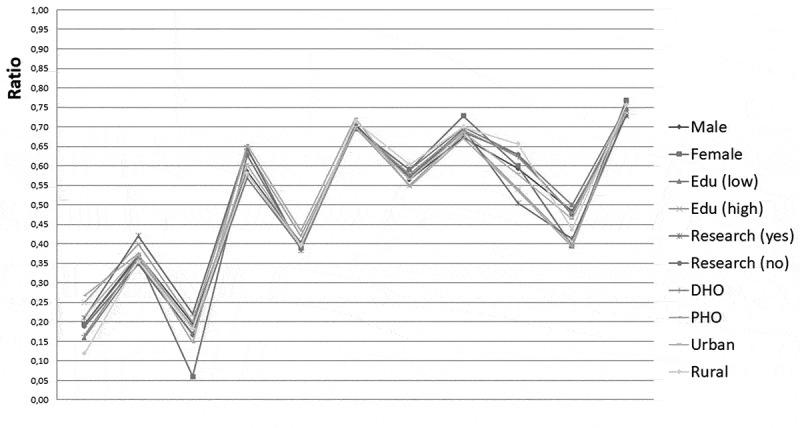
Research needs expressed in ratio by subgroup. On the x-as presents the ratio of how often of the total a topic was prioritised over another. 1=road traffic accidents, 2=CDs, 3=mental health, 4=MCH, 5=irrational drug use, 6=health system, 7=health education, 8=service provision, 9=sexual health, 10=NCDs, 11=health-seeking behaviour.

Table 2 shows the ranking of research domains as a result of the prioritisation exercise. Research into health-seeking behaviour was ranked highest, followed by health systems research. Mental health research was given least priority. No statistical differences were found between urban/rural provinces. We did find significant (p < 0.05) differences in ranking order based on educational level. Prioritisation by group is illustrated in Figure 2. It is quite apparent that the ratio of how often of the total a topic was prioritised over another was quite similar between groups.

**Table 2.** Research domains and avenues Phase 3.

Rank	Research domain avenues are presented underneath	Dominance*	Count**	Ratio***
1	*Health seeking behaviour* To understand underlying mechanisms causing underutilisation of services	30	999	0.74
2	*Health system research* Research related to strengthening the health system with a focus on health finance, health information systems, and effective human resource management	12	949	0.70
3	*Health service provision* Research related to improving service provision, its accessibility, acceptability and high quality of care.	3	930	0.69
4	*Mother and child health (MCH)* Research related to reducing mother and child disease burden during pregnancy, childbirth and the post-partum period, including improvement of immunisation coverage and nutritional status; the first 1000 days.	7	809	0.60
5	*Sexual & reproductive health* Research into sexual and reproductive health access, quality and information sharing, with a focus on young people.	4	802	0.59
6	*Health education* Research related to effective strategies to induce behavioural change.	1	773	0.57
7	*Non-communicable diseases (NCDs)* Research related to development of strategies to prevent and control NCDs, with a focus on diabetes, CVD, cancer and COPD	2	618	0.46
8	*Irrational drug use* Research related to reducing the high prevalence of irrational drug use and self-medication.	1	537	0.40
9	*Communicable diseases (CDs)* Research with a focus on communicable diseases with a focus on Dengue, Malaria, HIV, TB and Neglected Tropical diseases.	1	499	0.37
10	*Road traffic accidents* Research into strategies to prevent road traffic accidents	2	263	0.19
11	*Mental health* Research which examines the currently highly neglected field of mental health; how to provide high quality mental healthcare	0	246	0.18
		63	7425	-

## Phase 4: exploring research questions

After the ranking exercise, the research domains were set, but the avenues and questions to specify the agenda needed further elaboration and ranking. Fifteen interviews with experts on the interface of research and policy in Lao PDR were held. Experts were recruited through the network of the LaoTPHI/MoH the personal network of researchers involved in the stud and through snowballing. Participants had expertise in at least one of the 11 research domains and were asked questions about the research questions specific to their health expertise; they were not, however, limited to that topic in providing ideas. Additionally, interviewees were asked the reasons/criteria for making a particular question a priority. Interviews were recorded, transcribed, and coded. Research avenues and questions were listed and analysed using an inductive approach and linked to an already existing research domain. It appeared that research avenues needed further categorisation. Within avenues, we have selected topics (see Table 3 for examples). Questions were formulated in various ways, we have taken the liberty of re-formulation of questions. As an example, within health-seeking behaviour, information sharing was one of the avenues, questions under this avenue were; What conditions or illnesses do people seek information for?; Why do people *not* use health services, particularly in remote areas and among certain groups?; How to increase information-sharing of the benefits of health services?; How to increase the information-sharing about the health benefits of traditional medicine practices?; How to increase the reach of health information and the ability for individuals to act upon the information? Further example questions are presented in Table 3.

**Table 3.** The ranked public health research agenda; domains, avenues and one example questions.

Rank & Domain	Research avenues		Research questions	Delphi rank Round 1 & 2	
	**Larger avenue**	**Topics within avenues**		**1**	**2**
1 Health seeking behaviour	To understand underlying mechanisms causing under-utilisation of health services and how to improve people’s health seeking behaviour across Laos.	**Information sharing**	What conditions or illnesses do people seek information for?	**2**	**1**
		**Availability**	Who are the decision-makers for health seeking behaviour of children’s health?	**1**	**2**
		Accessibility (Barriers: Language, Discrimination, Migration)	What are the health seeking behaviours of migrated people (who are involved in logging, mining, and military occupations)?	3	3
		Affordability	Do people seek alternative options of care than national health services? What is the prevalence of this use and why?	4	4
2 Health system research	To achieve effective human resource management	Human Resources (HR)	How to assess the capacity and quality of healthcare staff and the overall health system?	1	1
	To establish an independent and well-functioning health financing system	Health Financing	How to achieve continuous and sustainable government healthcare funding, without dependence on donors?	2	2
	To further establish and improve the health information system	Health Information System (HIS)	How do we ensure that people use health information?	3	3
3 Health service provision	To develop policies steering the quality of services	Quality of health services (Responsive, efficient and effective)	How to improve the quality of services and health facilities?	1	1
	To increase access to health services	Access to care	How can services be extended to rural and remote areas in a way that access to services by the rural population is ensured?	2	2
	To provide acceptable health services	Acceptability of care	What are barriers for access to quality of services for people with disabilities?	3	3
4 Mother and child health (MCH)	To reduce neonatal mortality, under-5, and maternal mortality rate (MMR)	Ante-natal care (ANC) and Maternal care	Why pregnant women do not regularly come to the facilities for ANC?	5	
					1
		Neo-natal mortality	Why do people not give birth using skilled birth attendants?	2	2
	To increase immunisation coverage	Immunisation	What are the traditional perceptions on immunisation in different ethnic groups?	1	3
	To develop interventions with the aim to reduce malnutrition	Nutrition	Why do some communities not initiate early breast feeding?	3	4
	To reduce neonatal mortality, under-5, and maternal mortality rate	Children under-5 mortality	Even with antibiotics available, why are children dying of antibiotic preventable diseases?	4	5
5 Sexual health	To provide appropriate information and education to adults and young adults on sexual health topics	Sexual health education	What sexual health education is needed among adolescents and young adults?	2	1
		HIV	What is the prevalence of HIV/Aids in young people, people in remote areas, people living near the borders of China/Thailand/Cambodia/Vietnam, and Laos?	1	2
		Unintended pregnancy and unsafe abortion	What are the traditional practices in different ethnic groups and how do these relate to pregnancy at young age?	3	3
		Migration and sexual health	What is the impact of migration on the rates and prevalence of HIV?	4	4
6 Health education	To establish a health education system that facilitates behaviour change	Health education training	What are the training needs to better understand how people learn differently [in context of health education]?	2	1
		Prevention and promotion education	How to provide effective health education supporting behaviour change?	3	2
		Health education communication for different contexts	How to more effectively reach different audiences about health education?	1	3
7 Non-communicable diseases (NCDs)	To develop strategies to prevent and control NCDs	Diabetes	What are effective prevention measures to reduce the incidence of Diabetes?	1	1
		Heart disease (e.g. coronary heart disease, stroke)	What are effective prevention measures to reduce the incidence of heart disease?	2	2
		Cancer	What are effective prevention measures to reduce the incidence of different cancers? *	3	3
		Chronic obstructive pulmonary diseases [COPD] (related to smoking)	What are effective prevention measures to reduce the incidence of COPD?	4	4
		Disabilities	How could disabilities be diagnosed earlier and rectified against long-term affect?	5	5
8 Irrational drug use	To develop policies to reduce irrational drug use	Irrational drug use behaviour and education	What practices among healthcare professionals and patients contribute to drug resistance?	1	1
		Drug use and mental health	Why are young people using and becoming addicted to narcotics?	2	2
		Poorly prescribed drugs	How does the lack of prescriptions on drugs effect drug use behaviour?	3	3
9 Communicable diseases (CDs)	To develop interventions reducing the prevalence of Dengue fever	Dengue	How to more effectively prevent Dengue?	1	1
	To develop interventions reducing the prevalence of TB	Tuberculosis (TB)	How to more effectively prevent, detect, and treat TB?	2	2
	To develop interventions to eliminate malaria	Malaria	How to more effectively prevent, detect, and treat Malaria?	3	3
	To develop effective interventions to prevent the spread of HIV/AIDS	HIV/Aids and Multi-drug resistance	What is the impact of anti-viral resistance to HIV drugs?	4	4
	To develop effective interventions to prevent the spread of other CDs and NTDs	Communication about other CDs	How to improve the communication of how CDs are transmitted, especially in remote areas?	5	5
10 Road traffic accidents	To develop strategies to prevent road traffic accidents	Road traffic laws	How to enforce the road traffic law?	1	1
		Driver behaviour and effects	How to improve driver safety?	2	2
		Road infrastructure	How road infrastructure causes road traffic accidents?	3	3
		Mental health and road traffic accidents	What is the relationship between mental health and road traffic accidents?	4	4
11 Mental health	To examine how to provide high quality mental healthcare and further understand the current situation of mental health in Laos.	Depression (Postpartum depression	What are the incidence rates of suicide, amongst the national population?	2	1
		Diagnosis and services for mental health	How can services be adapted and extended to better meet the needs of children and young people?	3	2
		Perceptions of mental health conditions	What are the perceptions of citizens with regard to mental health in general, and treatment specifically?	1	3

## Phase 5: prioritisation of research avenues

The aim of this Phase was to inquire about relevant questions, rank avenues and if needed amend them and build consensus using a two-round Delphi method. A total of 33 participants joined the first round of the Delphi study and 22 in the second round. In accordance with Delbecq, Van de Ven, and Gustafson (1975 cited in Hsu, 2007), top management decision-makers, who will utilise the outcomes of the Delphi study, as well as health researchers and professionals were included [22]. The participants were selected based on their expertise in one or more of the 11 domains and their ability to reflect on other domains and were active in the public health domain in Lao PDR for 20 years or more. Researchers that had experience in sexual and reproductive health (12) and communicable diseases (10) were most frequent. Only one respondent had explicit experience in mental health.

The Delphi survey was administered online using the Survey Monkey tool. Round 1 asked participants to add and rank the avenues in each of the 11 health domains. We did not ask them to rank individual questions, but instead to rank the avenues based on the questions within the avenue or relevant questions for the avenue they wanted to add; individual ranking of questions within avenues for each of the domains would have been too laborious. Participants also provided weights to prioritisation criteria for questions, which was utilised in Round 2. Space was offered to add additional questions and their reasons for ranking number 1. Results from Round 1 were analysed and shared back to the group in Round 2.

Round 2 involved a Multi-Criteria Decision Analysis (MCDA). Participants were asked to prioritise research avenues based on questions on each of the four criteria established in Phase four of the study, and approved by respondents in Round 1 of the Delphi: 1) Local burden of the problem; 2) Research is in-line with government priorities; 3) Expected impact of the research; and 4) Feasibility of research application. Participants could give each avenue (representing the research questions within) points on a scale from 1 to 3. The mean of each avenue was then multiplied by the weight of each criterion to give the ranking for Round 2. The weight of each criterion was decided by participants’ ranking of the criteria in Round 1. This enabled an MCDA to be performed with weighted criteria. Additionally, in each health theme, the participants were shown the ranking from Round 1 and were asked to say if they disagreed with the ranking. This offered space for opinion sharing and consensus building. Results from Delphi Rounds were analysed through Survey Monkey, Excel, and STATA 15.1. Much consensus was observed within and between Delphi Rounds. None of the participants amended the avenues. Table 3 presents the outcome of cumulative Phases. We have provided one example question per avenue.

## Phase 6: Dialogue and planning for action

**Figure 3.**
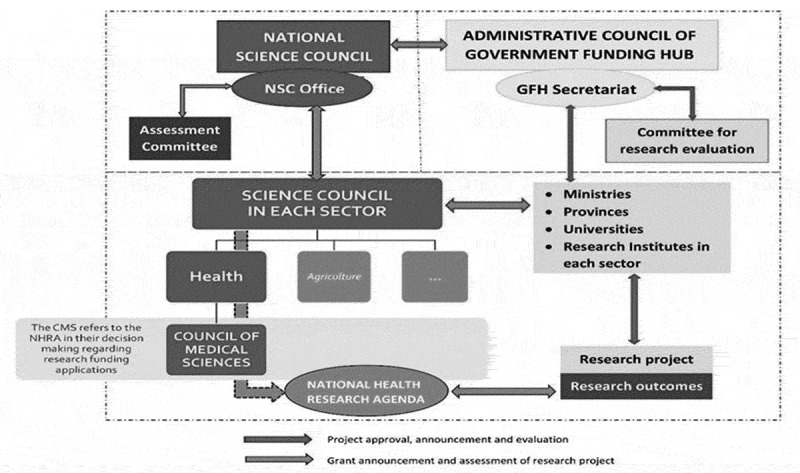
Organogram of how the research agenda is embedded within the MoH. On the left side in blue the governmental bodies that decide and approve research are presented. On the right side in yellow the organisations that take up/conduct the research are presented. The research agenda is informed by research outcomes, and is used by the medical ethical committee to evaluate research. Research outcomes have been given the colour purple to indicate where research and practice meet each other.

A half-day dialogue meeting was organised at Lao TPHI to finalise the agenda and improve the transparency of the decision-making process and to confirm and reflect upon the research agenda. In addition, a framework for integrating the agenda was discussed. A total of 45 stakeholders joined the discussion, including members of the Council of Medical Sciences, senior policymakers, senior health researchers, and representatives of donor agencies and NGOs (including many participants from Phases four and five). The session involved a ‘live’ prioritisation exercise using the programme Mentimeter. Each participant was given their own tablet with the Mentimeter-app installed. Participants worked in groups to discuss the research questions at their table, and their reasons for prioritisation. Note takers were present at each table to capture key discussion points and missing research questions. A plenary discussion was also organised in which the application of the agenda was reviewed. Three main points came forward from the dialogue. First, the conversation between participants confirmed that all of the research questions presented were important and all should be included in the agenda. Participants indicated that although the agenda is long, diverse, and probably not exhaustive, there is a need for it, and that the agenda should be used as a ‘living document’, and adapted when new research needs emerge. Any additional questions that were suggested during the meeting were collated, where possible, and were added to the full agenda of research questions. Second, participants agreed the agenda should be used as tool and reference in the development of health research in Lao PDR, and should continue to evolve with the changing context. The government of Lao PDR, in particular the Members of the Council of Medical Sciences, should use the agenda to steer national and donor funding towards national priorities. The implementation model is illustrated in Figure 3. Third, the prioritisation process itself, with all stages, was an important tool to engage diverse stakeholders, widen opinion, and create a ‘highly motivating experience for participants’ [23]. During the dialogue, and also in earlier Phases, it was evident that the need for the research priorities, consensus with the current ranking, and the ambition to institutionalise the tool were shared. In Table 4 we illustrate the increased awareness with some exemplary quotes from respondents.

**Table 4.** Quotes that illustrate the need for the tool.

*‘We need to adopt the use the agenda to make sure we do the right research’* (Phase six: member of the science council) *‘This is the dilemma in all of Laos. We are doing research because donors are interested in topics. It is not our own research. That is the situation now. … This study makes us aware’* (Phase four: Policymaker at the national level) *‘The agenda ranking is good, we need to focus on the health system … if [we] provide good quality of service, people will accept and use’ (Phase six, policy dialogue table notes)*. *‘In terms of public health research, I think it is quite difficult because many people are doing that but they are usually doing on their own, focussing on their disease. Sometimes they discuss when they meet in other meetings but often not. Different organisations, different teams have their own funding. Or have their own link with external funders. And you may know that sometimes, we done research but research results are not applied, are not used for policy-making. This is what we need to improve. What we call knowledge-transfer or policy brief or translation of research in to health policies. I think under the Learn project and with this agenda they are doing that.’* (Phase four: senior researcher and administrator at the UHS)

## Discussion

The approach used led to a comprehensive, inclusive, public health research agenda for Lao PDR to realise better informed health policies and better health programs, at the same time it improved institutional capacity of the MoH to steer and align research. This agenda fulfils one of the six key components of *the Strategy on Promotion and Management of Health Research 2015–2020* [6], identifying research priorities. The agenda, and how it is embedded in research funding appraisal processes, improves capacity of the MoH to steer research towards national priorities. This, in turn, contributes to generating evidence for policy making and implementation.

Our findings reveal that health policymakers and practitioners within the country prioritised research on health-seeking behaviour, followed by research to improve the health information system. The least value was ascribed to research strengthening mental health services. Researchers, senior policy makers and staff of international organisations prioritised questions and topics within the larger themes. The agenda itself improved institutional capacity for addressing priority research topics that can contribute to evidence-informed policies. The reflection on research priorities also increased awareness within the Lao health research and policy community for evidence informed approaches.

Questions on the agenda appear to be more action-orientated, inspired by a desire to understand the problems better, so that immediate improvements to services and interventions can be made. Questions related to research focussing on new discovery were limited. This may be a result of the restricted research capacity or of the nature of a ‘public’ health agenda which is fundamentally faced with the more immediate challenges of the nation, rather than fundamental research [24]. The involvement of policymakers who strive for short-term, pragmatic, goals could also be a reason for the problem-solving focus of research questions [25]. This can again be related to the short-term focus of health policymakers [26].

In *the strategy on Promotion and Management of Health Research 2015–2020* [6], broad research priorities were identified based on the MDGs. The domains in our agenda cover all these topics, but also addresses new areas for research. For example, the most pressing need identified in this study – research to understand why people are not using health services – had not been included as a national prioritised concern before [6]. Additionally, *the* strategy did not go to such detail as formulating avenues and questions. This is a clear addition to current research priorities. Furthermore, current research predominately focusses on communicable diseases, (mal)nutrition and reproductive health. This agenda confirms these priorities are important, yet more emphasis should be put on health system-oriented research priorities and NCDs. The expressed priority is also reflected in the changing burden of disease in Lao PDR. Between 2005 and 2016, the number of DALYs of the top 5 NCDs increased up to 40% [27]. Although the priorities are in line with the burden of disease, in practice research into NCDs and how the health system can address NCDs remains in its infancy in Lao PDR. Furthermore, none of the participants in the national survey prioritised mental health above any other topic. This may reflect the current lack of mental health services and stigma associated with it, it does not reflect the actual burden of disease attributed to mental health and the need for mental health services. Which is likely to be high, but neglected, as in other LMICs [28].

In the policy dialogue, the implementation model (see Figure 3) was decided upon. Currently, the Medical Research Council adopted the agenda in the appraisal of governmental research proposals. Proposals will be assessed on its relevance (partly) based on the agenda. In addition, donor-funded research should explicitly state how it is aligned to the agenda or why the proposed study is of importance but not on the agenda. This will be assessed as part of the ethical approval process. In addition, it is projected that the Lao TPHI will evaluate and update the agenda on bi-yearly basis to keep the agenda relevant as the context changes and new evidence is available. The implementation of the agenda can be hampered by barriers in the interpretative priority-setting process, ‘vertical budget silos, vested interests, political dominance, no real ability for change, and misalignment of incentives’ [29].

## Process and limitations

The inclusive process, with respondents from various governmental, research and civil society organisations across the public health domain, ensured that the agenda is comprehensive and implementation is well supported by the community. In that sense our process followed Viergever [1] and Abma & Broerse [12] to make our agenda informative and supported. The input from researchers from various sectors and policymakers from more than three quarters of all districts of Lao PDR makes the agenda generalisable to the country. Our process included more individuals form various perspectives than in most agenda setting processes in LMIC [10]. However, the selection of participants was conducted based on the network of the MoH. It is possible that experts with alternative views – which are less aligned to the government – are not included in the study. Regardless, we argue the variety of researchers and policymakers from different public health fields and geographical locations made the agenda robust.

Also, citizens outside of people working in the health sector did not have opportunities to contribute to the priority-setting, this could have led to new topics and different ranking [12]. The authors recommend to include end-users of health research in subsequent prioritisation actions. Pittens et al., Abma & Broerse provide interesting insights on how to do this and provide evidence that this leads to novel priorities and a different order [12,30,31].

## Conclusions

Over the course of the six-phase study, the health research agenda has been set, and capacity to develop an agenda, and institutional capacity to apply the agenda has been established. This study aimed to describe the outcome of the agenda, and the process to establish it. Regarding the outcome, findings revealed 11 prioritised research domains, 42 avenues and over 200 research questions. Health-seeking behaviour was ranked as the highest priority domain, followed by research to improve the health information system. Least value was ascribed to research strengthening mental health services. Having a better understanding of the research priorities in Lao PDR supports the MoH in general and the Medical Research Council, in particular, to assign limited resources for research. Resource allocation can now be justified based on the shared agenda which involved an extensive decision-making process and a diverse pool of stakeholders. This study further contributes to priority setting methodology. The process was inclusive and started with a broad inquiry and ended with a policy dialogue to disseminate and embed the agenda. The agenda-setting process strived to be transparent and extensive in nature so to ensure its purpose could gain both momentum and support within the wider health network in Lao PDR.

## Supplementary Material

Supplemental Material

## Paper context

This research was conducted within a broader capacity strengthening program for evidence informed public health policy making. A key element thereof was trying to guide research towards national and local policy needs. This study shows how research priorities can be selected in a participatory manner and thereby improving institutional capacity.
