# Supplementary material for: Developing a national health research agenda for Lao PDR: prioritising the research needs of stakeholders
Source: Glob Health Action. 2020 Aug 3;13(Suppl 2):1777000. doi: 10.1080/16549716.2020.1777000 (PMC7480602; doi:10.1080/16549716.2020.1777000)
Supplement: Supplemental Material [file ZGHA_A_1777000_SM5117.zip › Suplementary file full research agenda.pdf]

Rank & Domain	***Research avenues***		***Research questions***	***Delphi rank round 1&2***	
	**Larger avenue**	**Topics within avenues**		**1**	**2**
**1<br>Health seeking behaviour**	To understand underlying mechanisms causing under-utilisation of health services and how to improve people's health seeking behaviour across Laos.	1. **Information sharing**	What conditions or illnesses do people seek information for?<br>Why do people not use health services, particularly in remote areas and among certain groups?<br>How to increase information-sharing of the benefits of health services?<br>How to increase the information-sharing about the health benefits of traditional medicine practices?<br>How to increase the reach of health information and the ability for individuals to act upon the information (e.g. more vulnerable people)	**2**	**1**
		2. **Availability**	Who are the decision-makers for health seeking behaviour of children's health?<br>What are the culturally-sensitive reasons and other barriers (economic, location, lack of trust in health services, ethnicity) for why people do not use or access health services?<br>What is the health seeking behaviours of adolescents in the poorest groups in urban and rural in terms of care, prevention, and promotion of their health?<br>What are the health seeking behaviours of marginalised people, e.g. Lesbian Gay Bisexual Transgender (LBGT), non-Lao ethnic, the poorest groups in urban and rural in terms of care, prevention, and promotion of their health?<br>To what extent could we increase the use of health services, by rural people, through changing their behaviours?<br>What are the health seeking behaviours of marginalised people with disabilities?	**1**	**2**
		3. Accessibility (Barriers: Language, Discrimination, Migration)	What are the health seeking behaviours of migrated people (who are involved in logging, mining, and military occupations)?<br>How does domestic and international migration affect health seeking behaviour?<br>How does stigmatisation and discrimination affect people's behaviour to access services?<br>Why people use the health care services in neighbouring countries and what is the proportion of health seeking behaviours going to overseas services?<br>What barriers do persons with disabilities experience in accessing health services?*	3	3
		4. Affordability	Do people seek alternative options of care than national health services?<br>What is the prevalence of this use and why?	4	4
**2<br>Health system research**	To achieve effective human resource management	1. Human Resources (HR)	How to assess the capacity and quality of healthcare staff and the overall health system?<br>How can human resources be more effectively distributed in different regions?<br>Are jobs decided upon based on person's skill or based on what role is available at the facility?<br>Is donated hospital equipment being used effectively?<br>How to improve the capacity at District level health facilities? How does the training of medical practitioners differ across provinces? And why?<br>How to retain health care staff at the rural areas?<br>How could we improve the recruitment of staff according to the needs of the country?<br>How decisions are made about health policy and implementation at all levels?<br>How to improve the health system strengthening at all levels?	1	1
	To establish an independent and well-functioning health financing system	2. Health Financing	How to achieve continuous and sustainable government healthcare funding, without dependence on donors?<br>What is the feasibility and acceptability of universal health coverage?<br>What funding schemes are being used?<br>And are there any important finance mechanisms that are under-documented?	2	2
	To further establish and improve the health information system	3. Health Information System (HIS)	How do we ensure that people use health information?<br>Why are people not utilising the health information available?<br>What intervention can be used to improve communication and health information sharing at provincial/ district level?<br>What services are in place for the communication of the outbreak of diseases?<br>What routine information-sharing activities are taking place?<br>What routine information-sharing activities are effective?	3	3

**3 Health service provision**	To develop policies steering the quality of services	1. Quality of health services (Responsive, efficient and effective, equity and non-discrimination)	How to improve the quality of services and health facilities?<br>How to assess safety standards and the efficiency of staff in health facilities?<br>How to improve patient safety?<br>What is the validity and reliability of data entry around outbreak of diseases?<br>An assessment of the quality of drugs.<br>What are the percentages of actual components in the drugs?<br>How to maintain the quality of health services in health facilities? Who is the main person to be responsible for this task?<br>What are people's perceptions of traditional medicine?<br>What is the usage of traditional medicine in different regions of Laos?	1	1
	To increase access to health services	2. Access to care	How can services be extended to rural and remote areas in a way that access to services by the rural population is ensured?<br>How does the cost of travel affect access to health services?<br>Why do problems and inefficiencies happen in the supply chain of drugs? (E.g. stock outs, specifically Malaria)<br>Why are adolescent girls and boys not accessing health services, and what are the main barriers to their access?	2	2
	To provide acceptable health services	3. Acceptability of care	What are barriers for access to quality of services for people with disabilities?<br>How to better use volunteers to build capacity around health communication?<br>And what are the expectations of volunteers?<br>How to improve the performance of healthcare staff, including addressing negative attitudes towards patients?<br>How to ensure that health care staff are capable to respond to all patients (for example persons that have difference types of disabilities)?<br>What is the effect of stigmatization of mental health on acceptability of health services?	3	3
**4 Mother and child health (MCH)**	To reduce neonatal mortality, under-5, and maternal mortality rate (MMR)	1. Ante-natal care (ANC) and Maternal care	Why pregnant women do not regularly come to the facilities for ANC?<br>What are the causes of ANC dropout?<br>What are perceptions and attitudes of (young) families towards in-hospital delivery?<br>What are the cause of delays in seeking MCH services?<br>How to prevent delays of citizens in seeking MCH services?<br>Are mother's needs for ANC care being met?<br>What kind of Behaviour Change Communication (BCC) materials/messages should be used for new-born care (ANC, delivery, postnatal care (PNC)?	5	1
		2. Neo-natal mortality	Why do people not give birth using skilled birth attendants?<br>Is it necessary to have a midwife at every health facility?<br>What is the use of traditional birth attendants?<br>How to care for a new-born baby at home (especially immediately and within the 1st week of child delivery, within the different ethnic groups)?	2	2
	To increase immunization coverage	3. Immunization	What are the traditional perceptions on immunization in different ethnic groups?<br>What are the barriers around immunization in different ethnic groups?<br>What are the perceptions and attitudes of (young) parents towards vaccination programmes?	1	3
	To develop interventions with the aim to reduce malnutrition	4. Nutrition	Why do some communities not initiate early breast feeding?<br>What knowledge is known about the benefits of breast feeding in ethnic groups?<br>And the prevalence of alternative options?<br>What are the food practices and beliefs of communities in rural areas?<br>What are the eating habits for specific ethnic groups?<br>What cheap supplements are being used in child nutrition?<br>How can agriculture practices be adapted to produce more nutritionally-high food output?	3	4
	To reduce neonatal mortality, under-5, and maternal mortality rate (MMR)	5. Children under-5 mortality	Even with antibiotics available, why are children dying of antibiotic preventable diseases?<br>What about the health seeking behaviour of the parents for their children's health?<br>What is the accessibility of health services for parents in rural communities?<br>Why do people not seek the medicines at the local community level?<br>What is the main cause of death of new-borns and children under-5?	4	5

**5<br>Sexual health**	To provide appropriate information and education to adults and young adults on sexual health topics	1. Sexual health education	What sexual health education is needed among adolescents and young adults?<br>How to educate adolescents and young adults to encourage safe sex practices?<br>Is sexual health education accessible to different communities?<br>What interventions can be put in place to make sexual health information more accessible?<br>What are the motivations of girls to seek sexual health advice/ education?<br>What is the level of health literacy among Lao adolescent?<br>What is the best indicator to measure the improvement of sexual and reproductive health in adolescent?<br>How do we ensure that the young people reach sufficient information on SRH?<br>What is the self-capacity of young people to manage their sexually active life?<br>How to build a friendly environment for youth health?<br>How does the onset of menstruation affect school attendance?	2	1
		2. HIV	What is the prevalence of HIV/Aids in young people, people in remote areas, people living near the borders of China/ Thailand/ Cambodia/ Vietnam, and Laos?<br>Why are people living with HIV/Aids reluctant to disclose their status?<br>How does stigma and discrimination around HIV/Aids affect people's behaviour to get tested or receive treatment?<br>How do major infrastructure projects have effect on HIV rates and sexual health?	1	2
		3. Unintended pregnancy and unsafe abortion	What are the traditional practices in different ethnic groups and how do these relate to pregnancy at young age?<br>What are the social patterns/ behaviours around pornography in children?<br>What are the socio-economic factors at play in different ethnic communities that lead to early marriage?<br>What can be done to reduce teenage pregnancy and early marriage in Laos?	3	3
		4. Migration and sexual health	What is the impact of migration on the rates and prevalence of HIV?<br>How will the implementation of the new trans-national railways (through Laos) affect HIV rates?<br>What is the impact of migration from rural to urban on the perceptions of sexual health?	4	4
**6<br>Health education**	To establish a health education system that facilitates behaviour change	1. Health education training	What are the training needs to better understand how people learn differently [in context of health education]?<br>How to improve the skills of staff to communicate about health education?<br>How to improve the communication between practitioner and patient/ patient's family?<br>How to follow up and support supervision the person who received the Health education training?	2	1
		2. Prevention and promotion education	How to provide effective health education supporting behaviour change?<br>How can education be improved for lifestyle behaviour change, primarily aimed at the elderly?	3	2
		3. Health education communication for different contexts	How to more effectively reach different audiences about health education?<br>How to ensure health education can reach ethnic groups, people with disabilities, and rural communities?<br>What type of communication is needed to reach these ethnic groups?<br>What are the most effective approaches for the delivery of locally appropriate, affordable and high-quality health education for children and young people?<br>How can the method of peer-to-peer be used in different Lao contexts as a method of implementation of health education?	1	3
**7<br><br>Non-communicable diseases (NCDs)**	To develop strategies to prevent and control NCDs	1. Diabetes	What are effective prevention measures to reduce the incidence of Diabetes?<br>What is the diagnosis process look like Diabetes?<br>What health promotion measures are needed to encourage a healthy lifestyle, in relation to Diabetes, among citizens in urban areas?<br>How does the Lao health system support people affected by diabetes?<br>What is the relationship between diabetes and mental health?<br>What are the economic impacts of disabilities on households?<br>What are the patterns and prevalence, and causes, of overweight and obesity?	1	1
		2. Heart disease (e.g. coronary heart disease, stroke)	What are effective prevention measures to reduce the incidence of heart disease?<br>What is the diagnosis process look like heart disease?<br>What health promotion measures are needed to encourage a healthy lifestyle, in relation to heart disease, among citizens, particularly in urban areas?<br>How does the Lao health system support people affected by heart disease?<br>What are the risk factors for obesity, within the context of Lao PDR?	2	2

		3. Cancer	What are effective prevention measures to reduce the incidence of different cancers? *<br>What is the diagnosis process/ screening for early stage of the cancer? (e.g. breast cancer and cervical cancer)<br>How to more effectively support people affected by cancer?	3	3
		4. Chronic obstructive pulmonary diseases [COPD] (related to smoking)	What are effective prevention measures to reduce the incidence of COPD?<br>What are the causes of smoking-related diseases?<br>How much are people spending on smoking (the financial costs of smoking for the household)?<br>What is the cost of treatment, for the country, for smoking-related diseases?<br>How does the Lao health system support people affected by COPD?<br>What is the prevalence of smoking-related diseases?	4	4
		5. Disabilities	How could disabilities be diagnosed earlier and rectified against long-term affect?<br>What early interventions for the early-diagnosis of disabilities are possible in this context?<br>How does the Lao health system support people with disabilities?	5	5
**8**<br>**Irrational drug use**	To develop policies to reduce irrational drug use	1. Irrational drug use behaviour and education	What practices among healthcare professionals and patients contribute to drug resistance?<br>How to prevent irrational drug use among providers and patients?<br>How to improve education around irrational drug use?<br>How to revise or improve the current policy around irrational drug use?<br>How to change irrational drug prescription behaviour among medical doctors and strongly support for rational use of drug?<br>What policy requirements are needed for this?<br>How to assess rational drug use regularly at all health facilities?<br>How to ensure the national standard treatment guideline is updated and adhered to year by year?	1	1
		2. Drug use and mental health	Why are young people using and becoming addicted to narcotics?<br>What insights are known about the recovery in the rehabilitation centre for narcotics?	2	2
		3. Poorly prescribed drugs	How does the lack of prescriptions on drugs effect drug use behaviour?<br>What are the impacts of self-prescription behaviour?<br>An assessment of the quality of drugs being prescribed and the regulations in place.	3	3
**9**<br>**Communicable diseases (CDs)**	To develop interventions reducing the prevalence of Dengue fever	1. Dengue	How to more effectively prevent Dengue?<br>What are the current methods of promoting the prevention of Dengue?<br>What is the prevalence of Dengue fever?<br>What are effective interventions to control Dengue fever?<br>How are the various types of dengue fever distributed among society?	1	1
	To develop interventions reducing the prevalence of TB	2. Tuberculosis (TB)	How to more effectively prevent, detect, and treat TB?<br>What are the reasons we cannot detect more with the good testing equipment?<br>Why are people not adhering to the specific TB treatment?	2	2
	To develop interventions to eliminate malaria	3. Malaria	How to more effectively prevent, detect, and treat Malaria?<br>What are new solutions to eliminate Malaria?<br>What new interventions are needed to prevent Malaria?	3	3
	To develop effective interventions to prevent the spread of HIV/AIDS	4. HIV/Aids and Multi-drug resistance	What is the impact of anti-viral resistance to HIV drugs?<br>How to increase knowledge of HIV/Aids in remote areas?<br>How do we ensure HIV treatment is consistent?<br>How do different treatment regimes in neighbouring countries impact drug resistance?<br>How is migration affecting the prevalence and drug resistance of CDs?	4	4
	To develop effective interventions to prevent the spread of other CDs and NTDs	5. Communication about other CDs	How to improve the communication of how CDs are transmitted, especially in remote areas?<br>What are the preventative measures, response plans, and the pandemic preparedness in place for outbreak of CDs?<br>How can we advocate for more funding to NTDs?<br>How to improve the reporting of cases through an active and passive surveillance system?	5	5

**10**<br><br>**Road traffic accidents**	To develop strategies to prevent road traffic accidents	1. Road traffic laws	How to enforce the road traffic law?<br>What are the incidence rates of injuries/ disabilities in relation to lack of safety measures when driving?	1	1
		2. Driver behaviour and effects	How to improve driver safety?<br>What are drivers' behaviours in relation to safety measures on the road? (e.g. wearing helmet, adherence to speed limit)<br>What are the reasons for driver's unsafe behaviour?<br>Which populations are at risk?<br>How is this risk distributed among society?<br>What are the main risk factors for road traffic injuries among different road users?	2	2
		3. Road infrastructure	How road infrastructure causes road traffic accidents?<br>How can we improve the infrastructure of the road to reduce accidents?<br>An analysis on the demographics affected e.g. urban, rural, gender, age cohort, wealth status.<br>What is the quality of the rescue unit operation at the post-crash and during the transportation to the hospital/emergency?	3	3
		4. Mental health and road traffic accidents	What is the relationship between mental health and road traffic accidents?<br>What are the social determinants of mental health that affects road traffic accidents? [e.g. family problems]	4	4
**11**<br><br>**Mental health**	To examine how to provide high quality mental healthcare and further understand the current situation of mental health in Laos.	1. Depression (Postpartum depression	What are the incidence rates of suicide, amongst the national population?<br>What are the causes of depression in young adolescents?<br>What are the social determinants of depression?<br>What are the social impacts of postpartum depression for mothers?<br>What interventions could be effective in this context to tackle the prevalence of depression? *	2	1
		2. Diagnosis and services for mental health	How can services be adapted and extended to better meet the needs of children and young people?<br>How to increase the collaboration between practitioners and researchers in mental health research?<br>How to extend mental health services to reach more young people, rural communities, and ethnic groups?<br>What are the perceptions towards using traditional healers for mental health conditions?<br>What is the prevalence of mental disorders in the Lao PDR?<br>How to encourage caregivers to bring their children or young adolescent to access to health services for mental health?	3	2
		3. Perceptions of mental health conditions	What are the perceptions of citizens with regard to mental health in general, and treatment specifically?<br>How to improve the knowledge of mental health conditions, treatment, and perceptions for mental health staff?<br>What are the understandings between the difference between psycho-social disabilities and intellectual disabilities?<br>What discrimination behaviour is seen towards people with disabilities and mental health conditions?<br>What are the misunderstandings and miss-classifications of epilepsy as a mental health condition?<br>How is historical trauma transmitted through generations and how is it best treated? *<br>What mental health services would be accepted and used by people with mental health conditions?	1	3
