# Supplementary material for: Developing a national health research agenda for Lao PDR: prioritising the research needs of stakeholders
Source: Glob Health Action. 2020 Aug 3;13(Suppl 2):1777000. doi: 10.1080/16549716.2020.1777000 (PMC7480602; doi:10.1080/16549716.2020.1777000)
Supplement: Supplemental Material [file ZGHA_A_1777000_SM5117.zip › supplemantary to phase 3 Matrix for prioritising_.pdf]

	1. Research to reduce road traffic accidents	2. Research to control communicable diseases	3. Research to strengthen mental health services	4. Research to improve MCH	5. Research to reduce irrational drug use	6. Research to strengthen the health system	7. Research to provide better health education	8. Research to improve health service provision	9. Research to enhance sexual and reproductive knowledge	10. Research to respond to NCDs	11. Research to understand why people do not use health services
1. Research to reduce road traffic accidents											
2. Research to control communicable diseases	1 - 2										
3. Research to strengthen mental health services	1 - 3	2 - 3									
4. Research to improve MCH	1 - 4	2 - 4	3 - 4								
5. Research to reduce irrational drug use	1 - 5	2 - 5	3 - 5	4 - 5							
6. Research to strengthen the health system	1 – 6	2 - 6	3 - 6	4 - 6	5 - 6						
7. Research to provide better health education	1 - 7	2 - 7	3 - 7	4 - 7	5 - 7	6 - 7					
8. Research to improve health service provision	1 - 8	2 - 8	3 - 8	4 - 8	5 - 8	6 - 8	7 - 8				
9. Research to enhance sexual and reproductive knowledge	1 - 9	2 - 9	3 - 9	4 - 9	5 - 9	6 - 9	7 - 9	8 - 9			
10. Research to respond to NCDs	1 - 10	2 - 10	3 - 10	4 - 10	5 - 10	6 - 10	7 - 10	8 - 10	9 - 10		
11. Research to understand why people do not use health services	1 - 11	2 - 11	3 - 11	4 - 11	5 - 11	6 - 11	7 - 11	8 - 11	9 - 11	10 - 11	
